# Therapeutic Strategies and Drug Development for Vascular Cognitive Impairment

**DOI:** 10.1161/JAHA.117.005568

**Published:** 2017-05-05

**Authors:** Eric E. Smith, Alicja Cieslak, Philip Barber, Jerry Chen, Yu‐Wei Chen, Ida Donnini, Jodi D. Edwards, Richard Frayne, Thalia S. Field, Janka Hegedus, Victoria Hanganu, Zahinoor Ismail, Jamila Kanji, Makoto Nakajima, Raza Noor, Stefano Peca, Demetrios Sahlas, Mukul Sharma, Luciano A. Sposato, Richard H. Swartz, Charlotte Zerna, Sandra E. Black, Vladimir Hachinski

**Affiliations:** ^1^ University of Calgary Canada; ^2^ McMaster University Hamilton Canada; ^3^ National Taiwan University Hospital Taipei Taiwan; ^4^ Taiwan Landseed Hospital Taoyuan Taiwan; ^5^ University of Florence Italy; ^6^ University of Toronto Canada; ^7^ University of British Columbia Vancouver Canada; ^8^ Kumamoto University Kumamoto Japan; ^9^ Western University London Canada

**Keywords:** cerebrovascular disease, clinical trials, cognitive impairment, stroke, systematic review, vascular dementia, Cerebrovascular Disease/Stroke, Cognitive Impairment, Neuroprotectants, Secondary Prevention

## Introduction

Dementia is a large and growing health problem in developed and developing countries, with total costs approaching 1% of global gross domestic product, threatening the sustainability of healthcare systems.^1^ There are currently no disease‐modifying treatments for the most common cause of dementia, Alzheimer disease (AD). The second most common contributor to dementia risk is cerebrovascular disease.^2^ In contrast to AD, there is greater hope that vascular contributions to cognitive impairment can be prevented and treated. Recent evidence that the incidence of dementia is declining has prompted speculation, not yet confirmed, that this decline may be partly attributable to improved vascular care.^3^, ^4^

In this article, we review trial design and drug development for vascular cognitive impairment (VCI), focusing on symptomatic patients with vascular mild cognitive impairment (MCI) or dementia. First, we review axes along which vascular components of cognitive impairment can be addressed, including choice of trial population, trial intervention, and type of outcome. Second, we briefly review the pathophysiology of VCI, introducing the concept that trials may focus on disease modification, improving resilience, or enhancing cognition. Third, we systematically review prior drug trials for VCI patients according to drug class and trial size. Finally, we offer suggestions for methodological improvements for future trials.

## Trial Design Choices

**Figure 1:**
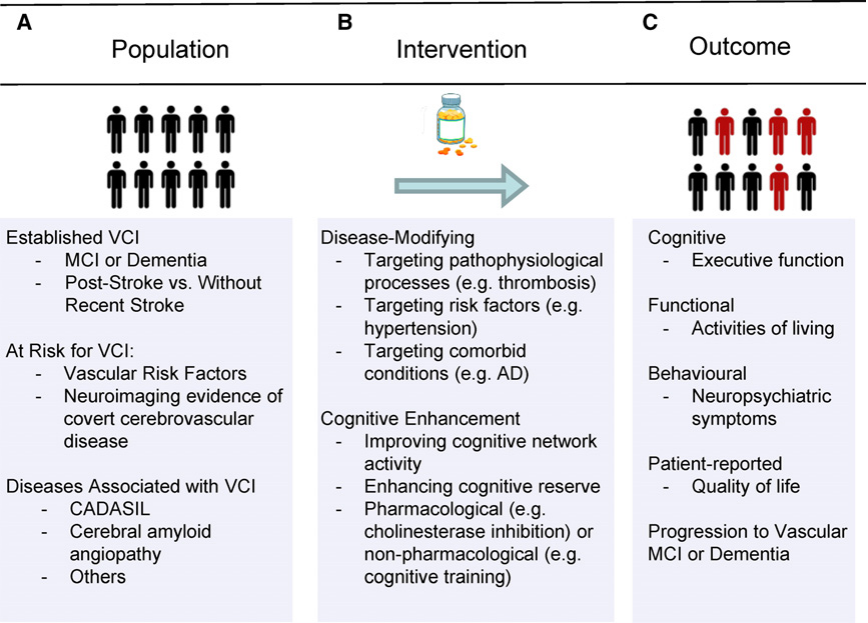
Trial design considerations categorized by population (A), intervention (B), and outcome (C). AD indicates Alzheimer disease; CADASIL, cerebral autosomal dominant arteriopathy with subcortical ischemic leukoencephalopathy; MCI, mild cognitive impairment; VCI, vascular cognitive impairment .

The effectiveness of treating vascular contributions to dementia would be best proved by randomized controlled trials (RCTs). Trials addressing the vascular contributions to cognitive impairment and neurodegeneration may target a specific population with VCI or at risk for VCI, a vascular intervention, or VCI as an outcome (Figure 1).

Patients with clinically diagnosed symptomatic VCI are an obvious target population (Figure 1A), and the primary focus of this review. The terms VCI, vascular contributions to cognitive impairment and dementia, vascular cognitive disorders, and vascular neurocognitive disorders all describe circumstances in which cognitive impairment or dementia is partly or primarily causally related to vascular disease. Recent clinical diagnostic criteria for VCI,^5^ vascular cognitive disorders,^6^ and vascular neurocognitive disorder^7^ are conceptually similar; henceforth, in this review we will use the term VCI to refer to these diagnostic entities. All of the VCI criteria rely on clinical evidence of a cognitive impairment syndrome (subdivided as MCI or dementia), clinical evidence of cerebrovascular disease (most commonly, history of stroke or neuroimaging evidence of severe clinically unapparent cerebrovascular disease), and a clinical judgment that the cerebrovascular disease is causing the cognitive impairment. The clearest circumstance is where there is a stroke that is immediately followed by new cognitive impairment; however, VCI attributable to “silent,” or more properly covert, cerebrovascular disease is actually more common. The term VCI also encompasses the common clinical scenario where cerebrovascular disease is accompanied by comorbid pathologies including AD and other neurodegenerative pathologies, which has been termed multiple etiology or “mixed” dementia.

VCI trials may also be defined based on application of an intervention to reduce vascular risk—whether to improve vascular function, prevent cerebrovascular disease, or prevent cerebrovascular injury (Figure 1B). The list of interventions that could be considered “vascular” in nature is quite broad when one considers all interventions whose effect may be wholly or partly mediated by improving vascular health. Examples could include blood pressure lowering, other forms of vascular risk factor reduction, or physical exercise. These interventions could be applied to the general population or to groups of patients at high risk for VCI. Such high‐risk groups could even include patients clinically diagnosed with other forms of neurodegeneration such as AD, given that multiple etiology dementia with a vascular component is the most common neuropathology of dementia,^8^ and that cerebrovascular function and disease have been hypothesized to promote AD pathology.^9^

Finally, VCI trials may be based on an expectation of preventing symptomatic VCI (Figure 1C). Many such trials define patient eligibility based on clinically defined cerebrovascular syndromes (eg, stroke) or radiological evidence of silent cerebrovascular disease, with or without cognitive impairment. This would include trials of cognitive rehabilitation after stroke where cognitive rehabilitation is provided to all stroke patients regardless of the baseline presence or absence of clinical VCI. Given the clinical difficulty in determining the causes of cognitive impairment and neuropathological evidence that the most common cause of dementia is not pure pathology in any form but rather multiple‐etiology dementia,^8^ it would be best for such trials to include all‐cause dementia or mixed dementia as outcomes, rather than restrict the outcome to clinically diagnosed pure vascular dementia.

## Pathophysiology of VCI: Implications for Therapeutic Development

Modern understanding of cerebrovascular function is based on the concept of a tightly integrated neurovascular unit.^10^ The role of the vasculature is to regulate perfusion to the tissue, responding to changes in neuronal activity, systemic perfusion pressure, and local changes to the chemical environment including partial pressure of carbon dioxide. Signaling by astrocytes, pericytes, and endothelium regulates arterial smooth muscle tone to deliver the appropriate amounts of oxygen and glucose to the brain.^10^ There is increasing evidence that the vasculature also plays a key role in waste product removal from the brain, including soluble abeta.^11^

When the vasculature becomes diseased, cerebrovascular injury to the brain may follow. Many, but not all, types of cerebrovascular injury may be detected using magnetic resonance imaging (MRI).^12^ Cerebral infarction and hemorrhage are the most destructive forms of injury, and may cause clinically evidence stroke syndromes. However, most cerebral infarctions are actually clinically unrecognized, or more accurately are associated with clinically hard to detect cognitive impairments.^13^ Thus, the estimated incidence and prevalence of MRI‐defined infarcts are more than 5‐fold higher than for symptomatic stroke.^14^ The prevalence of MRI‐invisible microinfarcts, which are tiny 50 to 200‐μm‐diameter infarcts below the spatial resolution of MRI but visible microscopically at autopsy, is even higher; they are detected neuropathologically in about 40% of all patients with dementia.^15^ As silent cerebral infarcts accrue over time, cognitive impairment worsens and risk for dementia increases.^16^

Other forms of detectable cerebrovascular injury include white matter lesions of presumed vascular origin, microbleeds, dilated perivascular spaces, and atrophy.^12^ White matter lesions of presumed vascular origin are readily detectable as white matter hyperintensity (WMH) on MRI, and are associated with increased risk for dementia. Although associated with vascular risk factors such as hypertension and cerebrovascular diseases such as cerebral autosomal dominant arteriopathy with subcortical ischemic leukoencephalopathy, the pathophysiology of WMH remains somewhat unclear and may be multifactorial.^17^

To treat vascular contributions to cognitive impairment and neurodegeneration, VCI therapeutic strategies could aim to reduce the build‐up of cerebrovascular injury, improve neurovascular unit function, or enhance cerebral resilience to injury. Disease‐modifying therapies (ie, those that reduce the accrual of cerebrovascular injury) hold the most promise for public health impact because they would treat VCI at its source. However, to date the most success has been obtained with symptomatic treatments (ie, those that improve function or cognitive resilience without affecting the rate of disease progression).

Reducing the build‐up of neurovascular injury could be accomplished by treating conventional vascular risk factors or by exploiting as‐yet unidentified, new molecular targets arising from research on the response of the neurovascular unit to vascular pathology. These trials will require longer durations or larger sample sizes to accrue sufficient numbers of new events such as incident infarcts.

Improving neurovascular unit function could restore better regulation of cerebral blood flow delivery to meet tissue metabolic needs. This restoration of homeostasis could theoretically improve function of ischemic neurovascular units in the short term, potentially requiring trials of only short duration to show improved outcomes. However, it remains controversial whether ischemia impairs cognition in the absence of infarction.^18^

Enhancing cerebral resilience to injury could be accomplished by making cells more resistant to hypoxia and ischemia (ie, neuroprotection), enhancing neuronal plasticity in the face of an injury, or increasing cognitive reserve so that future cerebrovascular brain injury is better tolerated. Enhancing cognitive reserve could potentially be accomplished by increasing neuronal plasticity, developing or retaining more numerous synaptic connections, or enhancing neuronal network function through interventions such as cognitive training, cognitive rehabilitation, lifestyle and behavior modification (eg, physical exercise), neurostimulation (eg, by transcranial direct current or magnetic stimulation), or drugs.

## Drug Development

Our review of drug development is based on a systematic review of all published RCTs in patients clinically diagnosed with VCI. RCTs in patients with VCI were identified by a search of ALOIS, an open access online database of all RCTs for cognitive impairment maintained by the Cochrane Collaboration Dementia and Cognitive Impairment Group (www.medicine.ox.ac.uk/alois). The “health status/diagnosis” field was searched on October 12, 2016, using keywords “vascular,” “infarct,” “stroke,” “ischemic,” “mixed,” “Binswanger,” and “circulation.” Two reviewers determined eligibility by consensus and abstracted data using standardized forms, and a third reviewer was added to resolve disagreements. RCTs were included if they described a randomized intervention to improve cognitive, behavioral, or functional outcomes in a patient population with VCI or mixed dementia with VCI diagnosed by standard criteria or by the study author's own criteria. Studies were excluded if outcomes were not reported separately for the patients with VCI or mixed dementia. Non‐English studies were included and read by native language speakers. Study quality was assessed using the Cochrane Risk of Bias Tool.^19^ More details on the review are available at the International Prospective Register of Systematic Reviews (PROSPERO ID CRD42016037958) at http://www.crd.york.ac.uk/PROSPERO. Institutional Review Board approval was not required because this was a systematic review.

**Figure 2:**
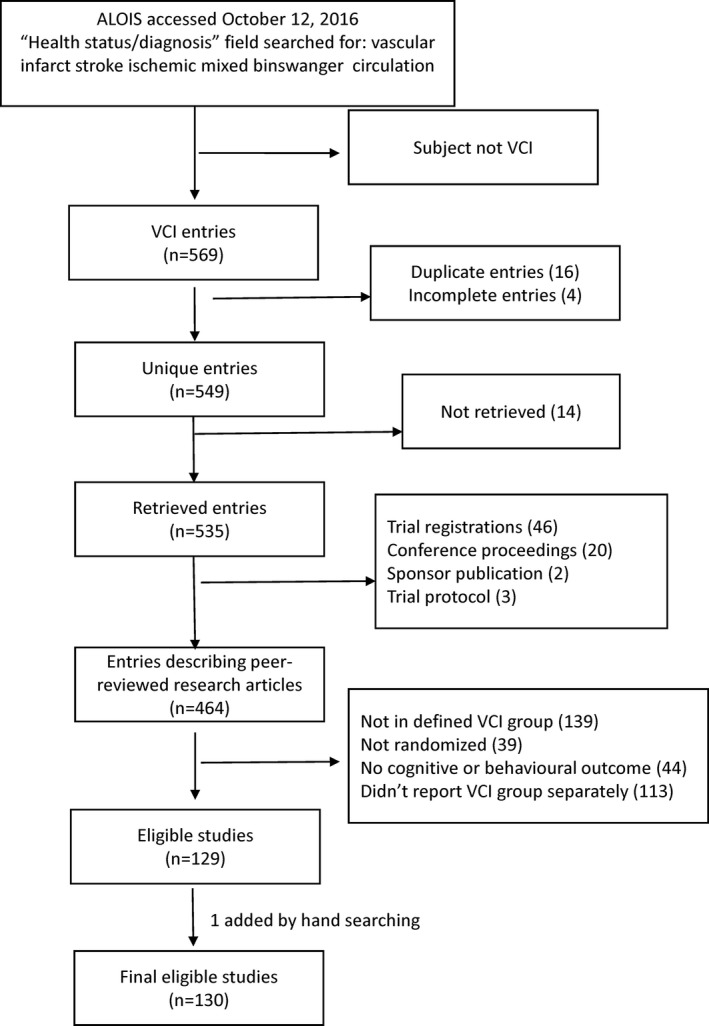
Flow chart showing study selection. ALOIS indicates the Cochrane Dementia and Cognitive Improvement Group's Specialized Register; VCI, vascular cognitive impairment.

**Figure 3:**
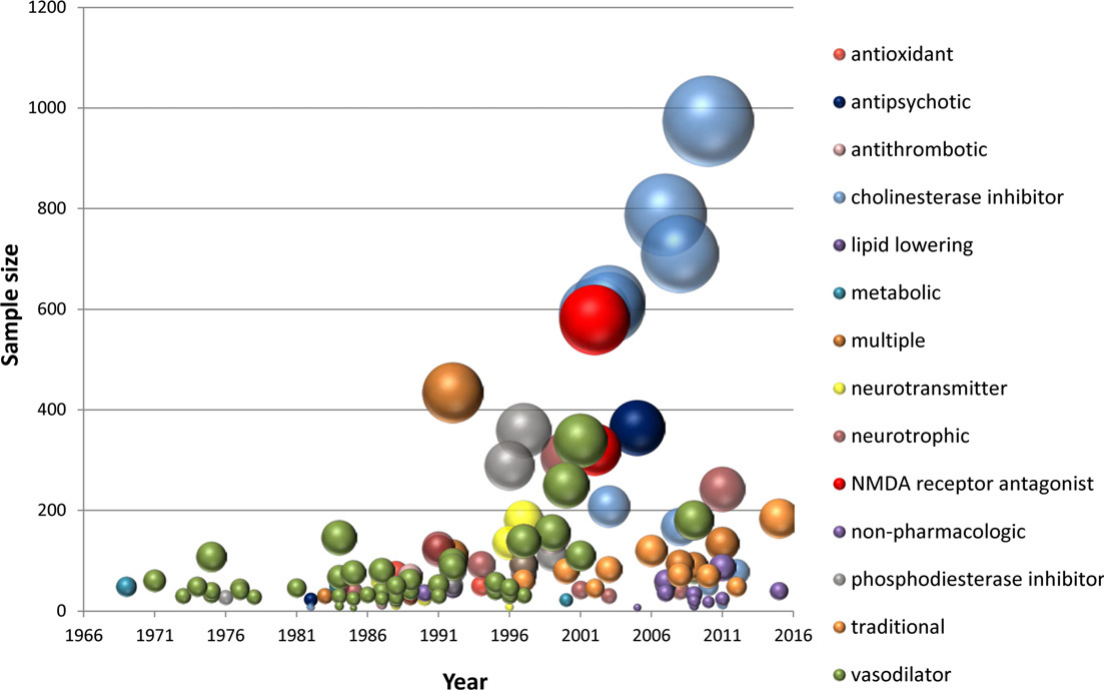
Published randomized controlled trials of pharmacological and nonpharmacological treatments for VCI. Data were abstracted from 130 eligible trials. The area of the bubble is linearly proportional to the total randomized sample size. Bubbles are color coded according to the presumed mechanism of action. “Multiple” indicates that the trial compared drugs or strategies with more than 1 mechanism, with or without an additional untreated control group. Nonpharmacological strategies included group therapy, acupuncture, and transcranial magnetic stimulation or direct current stimulation. NMDA, *N*‐methyl‐d‐aspartate.

We identified 130 previously published RCTs in which patients with VCI were treated with pharmacologic or nonpharmacologic interventions and followed for cognitive, behavioral, or functional outcomes (Figure 2). RCTs over time, classified by intervention type, are shown in Figure 3. Most RCTs included patients with vascular dementia, with only a few (7/130, 5.4%) restricted to patients with MCI or cognitively impaired but nondemented participants. Most included a placebo comparator (88/130, 68%). Most trials were of drugs; only a minority (14/130, 11%) tested nonpharmacological strategies. A diverse array of pathophysiological processes was targeted by drug types including vasodilators, neurotransmitter modulators, neurotrophic drugs, platelet antiaggregants, and antioxidants.

There were a larger number of individual trials reported in the 1980s and 1990s, but a larger number of patients randomized in the 2000s (Figure 3). Trials in the 1980s and 1990s predominantly tested drugs aimed at improving perfusion through vasodilation and other strategies reflecting the historical concept that cerebrovascular insufficiency could be reversed by increasing cerebral blood flow, while the later, larger‐scale trials predominantly tested cognitive‐enhancing cholinesterase inhibitors and memantine.

**Figure 4:**
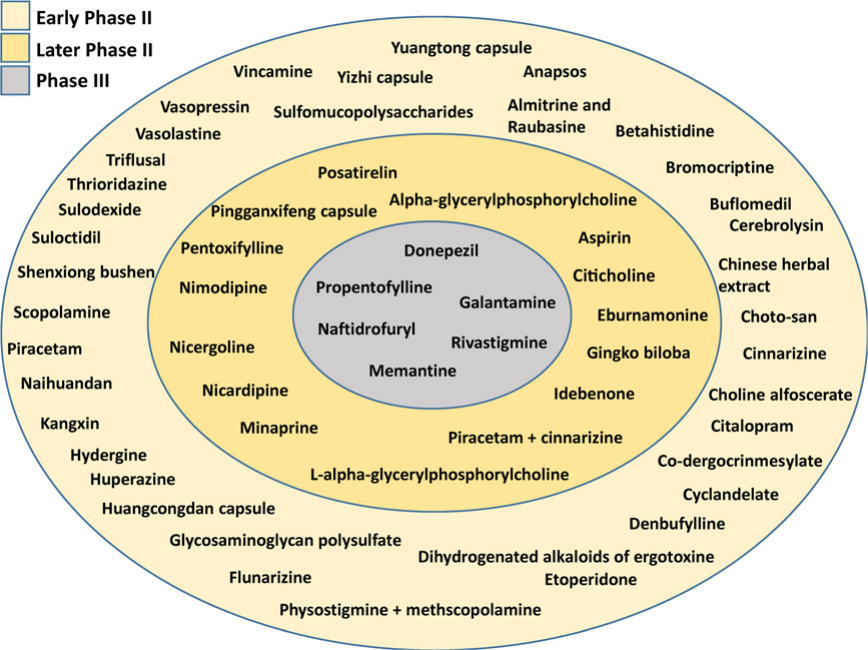
Drug development status of medications for patients with vascular cognitive impairment. Phase III was operationally defined retrospectively as randomized controlled trials with ≥300 participants, later phase II as ≥100 to 299 participants, and early phase II as <100 participants. Data from trials with untreated controls (mostly receiving a placebo) were used to generate the figure. A single large trial (>300 participants) of the calcium channel blockers nicardipine vs clentiazem was not included because there was no untreated control arm.

Individual drugs, categorized by maximum phase of development, are shown in Figure 4. Four drug classes proceeded to phase 3 RCTs: cholinesterase inhibitors, memantine, naftidrofuryl, and propentofylline.

The cholinesterase inhibitors reduce synaptic breakdown of the neurotransmitter acetylcholine, enhancing cholinergic transmission. They were originally developed in response to basic research showing a cholinergic deficit in patients with AD dementia. Subsequent work suggests that cerebrovascular injury also damages cholinergic pathways.^20^ Three cholinesterase inhibitors were tested in phase 3 trials of patients with vascular dementia or mixed dementia—donepezil,^21^, ^22^, ^23^ galantamine,^24^, ^25^ and rivastigmine^26^—and the results have been pooled and analyzed by the Cochrane collaboration.^27^, ^28^, ^29^ All of the phase 3 RCTs were 6 months in duration, and NINDS‐AIREN (National Institute of Neurological Disorders and Stroke and the Association Internationale pour la Recherche et l'Enseignement en Neurosciences) criteria^30^ were used to diagnose vascular dementia. All trials used the Alzheimer's Disease Assessment Scale‐Cognitive (ADAS‐Cog), or variants incorporating additional tests of executive function, as the primary cognitive measure, along with scales for global impression of change or activities of living. All of the trials reported positive effects on the ADAS‐Cog score (or variants); however, the absolute degree of benefit (≈2 points) was only about half of what was seen in AD trials (≈3–4 points) with the same durations and end points.^31^ Results on scales of global impression of change were mixed, with some RCTs reporting positive effects^27^ but others reporting no difference,^23^, ^25^, ^26^ and 1 trial reported no difference in AD Cooperative Study–Activities of Daily Living Inventory.^24^

Additionally, the cholinesterase inhibitor donepezil was studied in a RCT in patients with cerebral autosomal dominant arteriopathy with subcortical ischemic leukoencephalopathy and cognitive impairment or dementia.^32^ In this study, 168 patients with cerebral autosomal dominant arteriopathy with subcortical ischemic leukoencephalopathy were randomized to donepezil 10 mg per day or placebo for 18 weeks. The primary outcome was the V‐ADAS‐Cog. The difference in V‐ADAS‐Cog scores (0.4 points) was not significant, and donepezil treatment resulted in better cognitive test scores on only 1 of multiple secondary outcomes, the Trail‐Making test parts A and B.

Memantine is an *N*‐methyl‐d‐aspartate antagonist used as symptomatic therapy in moderate‐to‐severe AD. It has been tested in 2 trials of 28 weeks of treatment of patients with vascular dementia by NINDS‐AIREN criteria.^33^, ^34^ In 1 trial with 321 patients, memantine treatment resulted in better ADAS‐Cog scores (2.0 point difference) but no difference in the proportion with improvement or no change in the Clinician's Interview‐Based Impression of Change Plus Caregiver Input (60% versus 52%).^33^ In the second trial of 579 patients, memantine treatment resulted in better ADAS‐Cog scores (1.8 point difference, *P*<0.001) but no difference in the Clinician Global Impression of Change.^34^ The results of these 2 RCTs have been pooled and meta‐analyzed by the Cochrane group.^35^

Naftidrofuryl is a serotonin 5‐HT_2_ receptor antagonist with vasodilator properties, which has also been used as a treatment for peripheral claudication. It was tested in 4 RCTs in patients with vascular or mixed dementia with sample sizes of 6, 27, 108, and 339 participants.^36^, ^37^, ^38^, ^39^ The largest study was a 6‐month trial of 2 doses (400 mg per day and 600 mg per day) compared to placebo in patients meeting NINDS‐AIREN criteria for vascular dementia or mixed dementia.^39^ The primary outcome was the proportion of patients without any decline in either ADAS‐Cog or Sandoz Clinical Assessment Geriatric Scale at 6 months compared to baseline. The proportions without any decline were 58% for placebo, 75% for the 400‐mg dose (*P*=0.005 versus placebo), and 73% for the 600‐mg dose (*P*=0.02 versus placebo). Mean differences were not provided.

Propentofylline is a phosphodiesterase inhibitor and adenosine reuptake inhibitor with vasodilator properties. Results have been reported from 3 studies with 30, 90, and 359 participants.^40^, ^41^, ^42^ The largest study reported a pooled analysis of data from 4 individual RCTs with a total of 359 participants with vascular dementia diagnosed by NINDS‐AIREN criteria. These participants were randomized to propentofylline 600 mg per day versus placebo for either 6 or 12 months. Primary outcomes were not defined, but significant results at the final visit favoring propentofylline were reported for the Mini‐Mental State Examination (0.9‐point difference, *P*<0.01), Gottfries–Brain–Steen scale (3.5‐point difference, *P*<0.01), and Clinical Global Improvements (0.7 point difference, *P*<0.01) but not the syndrome short test (0.7‐point difference) or the Nurnberger–Alters–Beobachtungs–Skala for activities of living (−0.1 point difference). Methodological problems with the reporting of this pooled individual patient meta‐analysis include lack of detail on the conduct of the individual RCTs, lack of a specified primary outcome, and insufficient information on completeness of follow‐up. Baseline and follow‐up data from a subsequent phase 3 trial of propentofylline (MN‐305) in patients with possible or probable vascular dementia according to NINDS‐AIREN criteria were described in a subsequent review but the results of the placebo comparison were not provided^43^ and the sponsoring pharmaceutical company refused to provide the trial results to the Cochrane Collaboration.^44^ Consequently, the efficacy of propentofylline for vascular dementia is uncertain, and a risk for bias cannot be excluded in the published partial results.

In summary, the cholinesterase inhibitors advanced the farthest along the path to regulatory approval in the United States, Canada, and Europe but ultimately failed to receive approval. Concerns over the use of cholinesterase inhibitors for VCI have included the lower effect sizes compared to AD; the inconsistent benefits on clinical global impression, activities of daily living, and neuropsychiatric symptoms; and the possibility that cholinesterase inhibition may be improving symptoms of clinically unrecognized accompanying AD rather than VCI. Consensus groups convened by the American Heart Association and Canadian Stroke Best Practices recommend that cholinesterase inhibitors could be considered for use in VCI patients but with only intermediate‐grade evidence,^5^, ^45^ while the fourth Canadian Consensus Conference on the Diagnosis and Treatment of Dementia recommends against using cholinesterase inhibitors in VCI patients unless there is comorbid AD.^46^

Paradoxically, most of the RCTs we reviewed (109/130, 84%) claimed efficacy on 1 or more trial outcomes even though most did not progress to later phase studies and none have received regulatory approval. There are likely multiple reasons for this paradox. Most past trials were small (median randomized sample size 58, interquartile range 31–112) and short duration (median 84 days, 91/123 [74%] that reported duration were less than 24 weeks), many (78/130, 60%) did not analyze activities of living, and none analyzed progression from MCI to dementia. With these trial designs it is difficult to predict which candidate drugs are likely to have durable or disease‐modifying effects. Patient populations were heterogeneous with respect to diagnostic criteria and disease stage, particularly before publication of the NINDS‐AIREN criteria. Many trials did not require neuroimaging confirmation of cerebrovascular disease (67/130, 51%). Outdated trial designs make most of the previously published results vulnerable to bias, particularly those published before 2000. Commonly encountered methodological limitations included lack of prespecified primary outcomes, multiple hypothesis testing without appropriate adjustment, incomplete accounting of loss to follow‐up, and lack of intent‐to‐treat analysis, all of which predispose to false‐positive results.

A review of the clinicaltrials.gov trial registry (accessed June 6, 2016) revealed several ongoing or planned trials in patients with VCI. Among 10 RCTs that are listed as actively recruiting, trial interventions include cholinesterase inhibitors (NCT02660983, NCT02444637, NCT02098824), exercise (NCT02669394, NCT02550990), herbal medications (NCT02641886, NCT02453932), cognitive training (NCT02640716), hyperbaric oxygen (NCT02085330), and specialty vascular clinic care (NCT01924312).

## Nonpharmacological Treatments for VCI

A smaller number of trials (16/130, 12%) included nonpharmacological therapies as a comparator arm. Interventions tested were acupuncture or acupressure (3), electroacupuncture (2), reminiscence therapy (3), transcranial direct stimulation (3), sensory stimulation (1), transcranial magnetic stimulation (1), heparin‐induced extracorporeal low‐density lipoprotein precipitation (1), moxibustion (a traditional Asian medical therapy that involves burning the herb mugwort on the skin; 1), and carotid endarterectomy (1). Many of these trials originated in East Asia including China, Japan, and Korea (12/16). The studies were generally small (median 38 patients, range 7–180 patients) and of short duration (median 56 days; only 2 were longer than 90 days).

## New Opportunities for Trials in VCI

Our view is that the time is ripe for a new generation of VCI trials exploiting advances in diagnosis, outcome measurement, neuroimaging biomarkers, and trial design methodology, and a growing understanding of the pathobiology of vascular contributions to neurodegeneration (Table).

**Table 1:** Considerations for Improving the Quality of Trials in Patients With VCI

Consideration	Potential Strategies
Include clearly defined populations with VCI	Use modern VCI diagnostic criteria Require neuroimaging evidence of cerebrovascular disease as an inclusion criterion, with central adjudication When interactions with AD pathology are hypothesized, incorporate markers of the AD pathophysiological process
Use clearly defined clinically relevant end points	Include measures of cognitive function and activities of living Prespecify primary outcome and analysis plan Plan trial duration according to mechanism of effect: trials to prevent cerebrovascular disease progression may require longer duration than trials of cognitive/functional enhancement For trials of cognitive/functional enhancement, when appropriate include assessments of persistence of effect after active intervention ceases Include neuropsychological measures of executive function
Consider use of biomarkers where appropriate	When available, include biomarkers of on‐target intervention effects Further develop and validate biomarkers that may serve as monitoring biomarkers or surrogate end points in early phase trials
Improve quality of trial design and reporting	Adhere to Consolidated Standards of Reporting Trials (CONSORT)
Explore new potential therapeutic avenues	Develop and refine animal models of cerebral small vessel disease, cerebral amyloid angiopathy, large artery disease, and other vascular cognitive disorders Explore potential new therapeutic targets using translational approaches (eg, inflammation, oxidation, solute clearance, and restoring normal cerebral blood flow regulation)

AD indicates alzheimer disease; VCI, vascular cognitive impairment.

Trials should aim to improve cognition, daily function, and quality of life by targeting specific, prespecified mechanisms of impairment using appropriate diagnostic criteria and, when available, appropriate biomarkers for patient identification, target engagement, and outcomes. It is important to recognize that VCI is a syndrome, not a disease. Essentially any cerebrovascular disease that destroys brain can lead to VCI. Trial interventions should target not just a diagnostic syndrome but rather a specific mechanism of disease, brain injury, accommodation, or recovery in an appropriate population.^47^

### Patient Selection

Patients should be selected using modern diagnostic criteria with neuroimaging confirmation of cerebrovascular disease or related brain injury.^5^, ^6^, ^7^ The Standards for Reporting Vascular Changes on Neuroimaging provides consensus definitions for infarcts, WMHs of presumed vascular origin, microbleeds, enlarged perivascular spaces and brain atrophy, as well as suggestions for MRI acquisition protocols.^12^ Further validation work on the reliability of these criteria and standards in clinical and research practice would be welcome.

VCI caused by cerebral small vessel disease may be the most important target for future RCTs. Reducing the progression of cerebral small vessel disease and its impact on cognition and risk for dementia would produce large public health benefits. Neuropathology studies show that cerebral small vessel disease, predominantly in the absence of a clinical history of stroke, is found in most cases of dementia.^48^ It accounts for approximately one quarter to one third of the population‐attributable risk for dementia.^49^, ^50^ The majority of cerebral small vessel disease is caused by arteriolosclerosis because of aging, hypertension, and conventional vascular risk factors, while a minority is caused by vascular beta‐amyloid deposition (that is, cerebral amyloid angiopathy). The International Society for Vascular Behavioral and Cognitive Disorders has offered neuroimaging criteria for cerebral small vessel disease sufficient to cause vascular MCI or dementia.^6^ Further refinement and validation of neuroimaging criteria for vascular MCI and dementia would be helpful.

Vascular MCI may represent an ideal stage to intervene before accrual of irreversible disability. Because it is clinically difficult to discriminate MCI from very early dementia given that “generally mild functional impairment for complex tasks” is typically already apparent in MCI,^51^ it would also be reasonable to alternatively target early cognitive impairment based on cognitive concerns, low cognitive test scores, and preserved basic activities of living. Trials may also target pre‐MCI populations with neuroimaging evidence of cerebrovascular disease; however, definitive clinical trials will require larger sample size or longer durations to accrue enough clinical events, such as detectable cognitive decline or progression to dementia.

Trials must be designed with the awareness that most patients have multiple etiology dementia.^8^ Seeking patients with pure VCI for RCTs may be fruitless as few patients will be eligible, recruitment will be slow, and the trial results will not be generalizable to most patients with cognitive disorders. Instead, we suggest that eligibility should be based on ruling in VCI by neuroimaging without necessarily excluding the concomitant presence of other neuropathologies including AD. Whether to also assess for the presence or absence of AD pathology as a modifier of treatment effects depends on the nature of the intervention and the resources available. Cerebrospinal fluid and positron emission tomography markers of the AD pathophysiological process are expensive and invasive, respectively, and probably not feasible for most trials in VCI. Fortunately, assessment of AD pathology is not necessary to obtain unconfounded estimates of treatment effects because unmeasured AD pathology will be randomly distributed between study arms. However, the use of AD markers should still be considered when the trial intervention is expected to interact with AD pathology such that it may be more or less effective when AD pathology is present.

A criticism of some past trials is that improved outcomes may have been solely due to unmeasured effects on AD pathophysiology in patients with mixed dementia. Using vascular monitoring biomarkers could help to avoid this criticism by demonstrating that the intervention positively influenced vascular biomarker outcomes.

### Outcome Selection

Outcomes should be prespecified and should reflect the putative mechanisms targeted by the intervention. Definitive, phase 3 trial outcomes should include measures of both cognition and daily function, including activities of living.^52^ These outcomes should be sensitive to the effects of the intervention, disease stage, and duration of treatment. The US Food and Drug administration has outlined a path to accelerated preliminary approval of medications targeting early‐stage AD, before dementia, when beneficial effects on cognition are demonstrated.^52^ We propose that a similar approval strategy would also be appropriate for trials in VCI without dementia.

Cognitive outcomes should include measures of executive function. In particular, executive dysfunction is the hallmark of cerebral small vessel disease.^5^ Cognitive batteries designed to elicit the deficits in memory and orientation attributable to AD may lack sensitivity in VCI.^53^ The Canadian Stroke Network and National Institute of Neurological Disorders and Stroke have published harmonization standards for VCI research that include suggestions for neuropsychological test batteries of varying length based on criteria including psychometric qualities, portability, cost, ease of use, domain specificity, cross‐cultural capability, and lack of floor and ceiling effects.^54^

Secondary outcomes should be selected based on the trial population and presumed mechanism of action of the intervention, but may include neuropsychiatric symptoms, global clinical impression, caregiver burden, or healthcare utilization. Neuropsychiatric symptoms may appear early and be associated with risk for progressive cognitive decline or incident dementia.^55^ Apathy, depression, psychosis, and agitation are common neuropsychiatric symptoms in VCI.^6^

Trial duration should be sufficient to show durable effects. Six months is probably sufficient for definitive trials of cognitive‐enhancing interventions (as in the cholinesterase inhibitor trials) but longer durations will probably be required for trials aimed at preventing disease progression.

To date, no trials have aimed to prevent progression to dementia in patients with vascular MCI. However, trials to prevent progression to dementia may need to be large and of long duration. Two cohort studies have investigated the risk for progression to dementia in patients with VCI not demented. In the clinic‐based Canadian Cohort Study of Cognitive Impairment and Related Dementias (ACCORD), 10/25 (40%) of patients with VCI not demented converted to dementia after 2 years.^56^ In the population‐based Canadian Study on Health and Aging, 58 of 126 participants (46%) with VCI not demented progressed to dementia over 5 years of follow‐up.^57^ More contemporary, longitudinal studies are needed on the natural history of vascular MCI,^58^ including the rate of progression to dementia and risk factors for progression.

### Biomarkers

Whenever feasible, biomarkers should be used to identify target diseases and mechanisms and whether the intervention is acting on its target. Such biomarkers are especially useful in early‐phase trials to discriminate promising from less promising candidates to move forward to later‐phase trials. For example, MRI arterial spin label perfusion imaging would be a useful marker of the effect of agents designed to increase cerebral blood flow. Although biomarkers can take many forms, including genetics and fluid markers,^59^ neuroimaging currently appears to be the most promising method for detecting cerebrovascular injury that contributes to risk for VCI.^12^

Monitoring biomarkers can be used serially to detect changes in the degree or extent of disease.^60^ In longer‐duration trials aimed at preventing progression of cerebral small vessel disease, follow‐up MRI could identify intervention‐related differences in accrual of clinically hard to detect cerebrovascular disease including silent infarction and WMH progression. Because it can be measured quantitatively, differences in WMH progression and white matter tract connectivity can be detected with relatively small sample sizes.^61^, ^62^ WMH measurement has already been incorporated as a monitoring biomarker in several RCTs in cardiovascular disease.^63^, ^64^, ^65^, ^66^, ^67^ However, limitations of WMH as a biomarker include a relatively weak association with cognition,^62^ insensitivity to early microstructural changes associated with cerebrovascular disease,^68^ and lack of clarity with regard to its pathogenesis. For example, in the Action to Control Cardiovascular Risk in Diabetes Memory in Diabetes study (ACCORD‐MIND), intensive glucose control was associated with increased, not decreased WMH progression for unclear reasons.^65^ Newer candidate neuroimaging markers include microinfarcts, perivascular spaces, microbleeds, and diffusion tensor imaging of white matter microarchitecture and brain connectivity.^12^

To be considered a surrogate end point, a biomarker must be an indicator of pathogenic processes that strongly predict clinical response such that the biomarker change could substitute for a direct measure of how a patient feels, functions, or survives.^60^ Validation of a biomarker for this purpose requires strong data with a clear mechanistic rationale and strong evidence that the effect on the surrogate predicts clinical benefit.^69^ To date, there is no validated surrogate end point for VCI trials. Unfortunately, the association between WMH progression and progressive cognitive decline is probably too weak for it to be considered a surrogate end point.^61^, ^62^ However, this should not preclude the use of WMH progression as a monitoring biomarker to identify early changes in disease progression.

### Trial Design

The next generation of VCI trials should use modern designs including trial registration, prespecified primary outcomes, blinding (where possible), intent‐to‐treat analyses, and rigorous randomization and allocation concealment. After a period of privileged access by the trial investigators, de‐identified individual patient data should be uploaded to a repository for access by the entire research community.^70^ Adherence to harmonization standards for VCI research^54^ will facilitate individual patient data meta‐analysis. If patient subgroups are defined consistently using harmonized data elements, then patient information could be combined from multiple trials, amounting to a fairly large sample size that may tentatively identify subgroups with positive results. Trial reporting should adhere to Consolidated Standards of Reporting Trials.^71^

Ultimately, it is likely that VCI treatment will include a combination of adherence to conventional vascular risk reduction, lifestyle modification, cognitive enhancement, and potentially pharmacological treatment to ameliorate or restore pathobiological changes in the diseased neurovascular unit. Such treatment may be similar to the multifactorial intervention that successfully improved cognitive performance in the Finnish Geriatric Intervention Study to Prevent Cognitive Impairment and Disability study.^72^ Future trials in VCI patients should explore the efficacy of such multifaceted interventions, potentially using factorial designs to identify additive versus interactive effects of different treatment arms.

### New Targets for Treatment

Finally, future trials should exploit advances in the understanding of the pathobiology of the neurovascular unit. There is no clear consensus on the most promising molecular target: recent studies have investigated diverse pathophysiological mechanisms including vascular oxidative stress, inflammation, immune trafficking, blood–brain barrier permeability, vascular beta‐amyloid, neurovascular signaling, trophic coupling, perivascular and vascular solute clearance, and others.^10^ Animal models have been developed that reproduce specific aspects of human VCI syndromes and diseases including chronic forebrain ischemia, chronic hypertension, and cerebral amyloid angiopathy.^73^ These models are being used to screen drugs for beneficial effects. Further refinement of these models and the development of ex vivo experimental systems could in the future allow more efficient screening of drug libraries for effects on pathobiological mechanisms.

## Conclusions

There are no proven treatments to reduce the risk of progressive cognitive and functional decline in VCI patients. Cholinesterase inhibitors provide modest cognitive benefit but the effect appears to be less than that seen in AD patients with inconsistent findings on activities of living, physician and caregiver impression, and behavior. A large number of other compounds have been tested in small‐scale early‐phase RCTs but have not advanced to later‐phase trials. Promising candidates for advancement are difficult to discern because of trial methodological limitations that confer high risk for biased, false‐positive results. However, advances in diagnosis, neuroimaging, trial methods, and harmonization standards for VCI research suggest that there is more fertile ground for a new generation of trials to improve outcomes in VCI, the second most common contributor to dementia.

## Sources of Funding

This review was funded by the Canadian Consortium on Neurodegeneration and Aging Vascular Illness team, through a grant from the Canadian Institutes of Health Research.

## Disclosures

Dr Smith reports grants from the Canadian Institutes of Health Research Canadian Consortium on Neurodegeneration in Aging; Canadian Institutes of Health Research, Brain Canada, Canadian Partnership Against Cancer, and research contracts with McMaster University to provide MRI analysis services. Dr Black reports institutional grants from Pfizer, GE Healthcare, Eli Lilly, Elan/Transition Therapeutics, Roche, Cognoptix, Biogen, Lilly‐AVID, and personal fees from Pfizer, Eli Lilly, Boehringer Ingelheim, Novartis, Merck, Eisai, Medscape, and Biogen Idec. Dr Field reports grants and personal fees from Bayer Canada, personal fees from Pfizer, and grants from Boehringer Ingelheim Canada. Dr Sharma reports grants and personal fees from Boehringer Ingelheim and Bayer, grants from Bristol Myers Squibb, and other support from Daiichi Sankyo. Dr Swartz reports grants from Heart and Stroke Foundation of Canada, and other support from Canadian Partnership for Stroke Recovery. The other authors report no disclosures.
